# Teaching distinguishing semiological features improves diagnostic accuracy of seizure-like events by emergency physicians

**DOI:** 10.1186/s42466-022-00220-w

**Published:** 2022-11-14

**Authors:** Wenke Grönheit, Vanessa Behrens, Tatjana Liakina, Christoph Kellinghaus, Soheyl Noachtar, Stoyan Popkirov, Tim Wehner, Eva Brammen, Jörg Wellmer

**Affiliations:** 1 Ruhr-Epileptology, Department of Neurology, University Hospital Knappschaftskrankenhaus Bochum, Ruhr University Bochum, In der Schornau 23-25, 44892 Bochum, Germany; 2 Department of Neurology and Neurosurgery, Institute of Clinical Medicine, Faculty of Medicine, Vilnius University, Vilnius, Lithuania; 3 Department of Neurology, Klinikum Osnabrück GmbH, Am Finkenhügel 1, 49076 Osnabrück, Germany; 4 Department of Neurology, Ludwig-Maximilians-University Munich, Klinikum Großhadern, Marchioninistr. 15, 81377 Munich, Germany; 5 Department of Neurology, University Hospital Knappschaftskrankenhaus Bochum, Ruhr University Bochum, In der Schornau 23-25, 44892 Bochum, Germany; 6 Chrestos Institut, Chrestos Concept GmbH & Co. KG, Girardetstr. 1-5, 45131 Essen, Germany; 7 Department of Clinical Neuroscience, University of Gothenburg, Blå stråket 7, plan 3, Sahlgrenska, 41345 Göteborg, Sweden

**Keywords:** Semiological elements, Seizure mimics, Pre hospital medicine, Positive predictive value

## Abstract

**Background:**

Misdiagnosis of seizure-like events (SLE) in emergency situations is common. Here, we evaluate whether a single, video-based lesson highlighting distinguishing semiological features can improve the diagnostic accuracy of emergency physicians for epileptic seizures (ES), psychogenic non-epileptic seizures (PNES) and syncopes (SY).

**Methods:**

40 emergency physicians (24 anesthetists, nine surgeons and seven internal medicine specialists by primary specialty) participated in a prospective trial on the diagnostic accuracy of SLE. They assessed video-displayed SLE at two time points: before and after a lecture on distinguishing semiological features. In the lecture, semiological features were demonstrated using patient videos, some were acted by the instructor in addition. The increase in correct diagnoses and recognition of distinguishing semiological features were analyzed.

**Results:**

Before the lesson, 45% of 200 SLE-ratings were correct: 15% of SY (*n* = 40), 30% of PNES (*n* = 40), 59% of ES (*n* = 120, focal to bilateral tonic–clonic seizures (FBTCS) 87.5% (*n* = 40), focal impaired aware seizures (FIAS) 45% (*n* = 80)). Semiology teaching increased both the rate of correct diagnoses of SLE to overall 79% (*p* < 0.001) (ES 91% (*p* < 0.001), FBCTS 98% (n.s.), FIAS 88% (*p* < 0.001), PNES 88% (*p* < 0.001), SY 35% (*p* < 0.001)), and the number of recognized distinguishing semiological features. We identified several semiological features with high entity specific positive predictive values (> 0.8).

**Conclusions:**

A single 45-min video-based lesson highlighting distinguishing semiological features improves the diagnostic accuracy of ES, PNES and SY by emergency physicians. We expect that including this aspect into the curriculum of emergency physicians will lead to better individual patient treatment in pre-hospital medicine and more appropriate subsequent use of clinical resources.

**Supplementary Information:**

The online version contains supplementary material available at 10.1186/s42466-022-00220-w.

## Background

Epilepsy is one of the most common admission diagnoses in neurology [4, 21]. Suspected epileptic seizures (ES), including mimics such as psychogenic non-epileptic seizures (PNES) and syncope (SY), account for around 1% of all emergency hospital admissions [6, 17]. Amongst unscheduled neurological admissions, suspected seizures (seizure like events, SLE) account for 13–47% [6, 21]. In the city of Bochum, Germany, where this study was conducted, SLE account for about 7% of all emergency calls (data refer to 2014 and 2015) and 4.8% of in-patient admissions through our hospital emergency department (internal audit, 2014–2017).

Although physicians are so frequently confronted with ES and its mimics, misdiagnosis is common [7, 16, 19, 25]. Misdiagnosing an SLE can bias the further diagnostic and therapeutic process with potentially harmful medical and social consequences, and negative health economic implications [5, 12–14, 17, 19, 20, 23, 27].

Possible reasons for misdiagnoses of SLE are [3, 11]:

- ES, PNES and SY usually occur irregularly and infrequently and are often not witnessed by the diagnosing physician personally.
- Unstructured witness reports are often misleading.
- Once the event is over, there are rarely objective clinical or technical criteria that allow a diagnosis.

The most important instrument to correctly diagnose SLE is their precise semiology [2, 9, 15, 26]. Specialized epileptologists, for example, inquire thoroughly from patients and witnesses all symptoms occurring during the events. They attempt to understand the dynamics of movement patterns in patients with ictal motor symptoms, capture the spectrum of non-motor symptoms and analyze the sequence in which these symptoms occur (confirming or violating neuroanatomical logic). The duration of events and potentially provoking factors provide additional hints. Recognition of specific semiological features in many cases allows a definite diagnosis without the need for technical examinations [1].

The closer to the event semiological information can be gained, the more reliable it is likely to be. Therefore, providing paramedics and emergency physicians with knowledge of semiology could be a suitable strategy to reduce the rate of misdiagnoses of SLE [8, 22].

Previous studies have shown that lessons using videos of real SLE can improve diagnostic accuracy of ES and PNES in medical students [22]. This encouraged us to develop and evaluate a video-based lecture tailored to the needs of emergency physicians who usually first encounter SLE. Furthermore, our analysis aimed to determine the value of individual semiological features in diagnostic decision-making (search for features with a high positive predictive value; [10]), which could inform future diagnostic algorithms.

## Methods

We conducted a prospective, single-blinded study assessing the ability of emergency physicians to correctly diagnose SLE before and after a lecture on distinguishing semiological SLE features. To understand if a potential learning effect is enduring, and second post-lesson evaluation was performed > 6 months later. The ethics committee of the Medical Faculty, Ruhr-University, Bochum approved the study (Reg. No. 5132–14).

All patients whose SLEs were shown had agreed to the presentation of their recordings in training sessions for medical personnel. Only in one video the face was pixelated at the request of the patient (video A1).

### Study participants

Study participants were physicians who already practice or seek to practice as emergency physicians in addition to their primary employment. This activity is voluntary, but in order to acquire or maintain the qualification, a curricular course on all topics of emergency medicine must be attended. The topic of differential diagnosis of SLE was not previously part of the course curriculum. In contrast to course attendance, participation in the study was voluntary.

Study participants were recruited from three parallel classes of the course with a total of 120 emergency physicians. All received the lecture on SLE. 40 attendees agreed to participate and were included in the study. We collected data about participants’ professional experience and their confidence to correctly diagnose SLE via a questionnaire.

### Content of teaching lesson

Net lesson time (i.e. exclusive of the study administrative part and the time participants had for watching and rating the study videos) was 45 min. The lesson systematically pointed out distinguishing characteristics of ES, PNES and SY that are reliably recognizable for non-specialists. A summary and characterization of taught semiological features is given in Additional file 1: Tables S1 and S2. They were shown in instructive video sequences; in addition, some features were acted by the lecturer (JW) with particular emphasis on different dynamics of motor phenomena. Two authors (WG and VB) confirmed that all semiological features needed to correctly diagnose SLEs in the study were taught during the lecture.

### Seizure videos for diagnostic evaluation

**Fig. 1:**
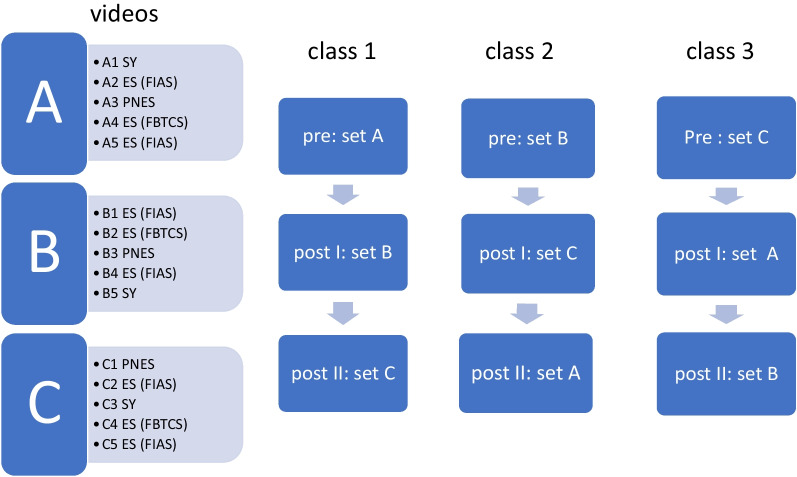
Sets of videos (A1-5, B1-5, C1-5) including the order of seizure-like event types and the time points (pre, post I and post II lesson) they were presented

15 videos were selected from video-EEG-recordings and from smartphone videos taken by relatives and divided in 3 sets of 5 videos each. Each set comprised three ES (one focal to bilateral tonic–clonic seizure, FBTCS, and two focal impaired awareness seizures, FIAS), one PNES and one SY. For detailed description of the presented SLE see Additional file 1: Tables S1 and S2. Before and after the lecture as well as 6–8 months after the course (if participants agreed) separate sets of videos were presented. Between parallel classes 1 to 3, the order of video sets was permuted (see Fig. 1).

The correct diagnoses of SLE shown in the videos (ground truth) was ensured either by diagnostic work-up (Video-EEG, ECG, result of a suggestive seizure induction; Popkirov et al., 2017, 2020) or, if this was not possible (3 cases of smartphone videos), by consensus of the Bochum epileptologists (JW, WG, TW) and two external epilepsy specialists (CK, SN).

To further ensure that the correct diagnosis of SLE presented to participants could be made based on semiology alone, four authors of the study (WG, JW, VB, TL) rated all videos with regard to the presence of distinguishing semiological features. In 14 out of 15 videos, they found that discrimination was clearly possible. These videos contained only features characteristic for one of the three entities (see Additional file 1: Table S1). One video however (B5) showing a 1 min segment of a syncopal loss of consciousness (following a venous puncture not visible on the video) contained features of ES and SY (repeated vocalization similar to a tonic vocalization which could be mistaken for epileptic tonic vocalization). Nevertheless, because of the real world character of this event, it remained part of the video set.

### Evaluation of events by participants

As shown in Fig. 1, participants watched one video set (either starting with set A, B or C) before their lesson. They were asked to write down the most likely diagnosis of the shown SLE and to note semiological features which they considered relevant for the diagnosis.

Directly after the lesson (post I) participants rated their second set of videos (B, C or A, see Fig. 1) in the same manner. Those participants who agreed to a post II evaluation ≥ 6 months after the lecture were visited by one of the authors (VB) at their work place to evaluate the last set of SLE videos on a laptop computer.

In either setting, each video was presented only once and participants had two minutes after each video to freely document semiological features and their diagnoses.

### Statistics

Descriptive statistics were used to evaluate the work biographical data of study participants. Analyses were carried out using R version 4.0.3.

### Primary endpoint

The learning effect of participants immediately after the lesson (pre vs. post I) was analyzed across all SLE and for individual entities (ES, FIAS, FBTCS, PNES, SY) using McNemar test with continuity correction. A *p*-value < 0.05 was considered statistically significant.

The follow up effect of training (post vs. post II) was evaluated using descriptive statistics.

### Secondary endpoints

The ability of participants to recognize distinctive semiological features within presented videos before and after the lesson was the secondary endpoint of this study. Not all taught features of table S1 were later assessed since features such as ‘behavioral arrest’ and ‘absent staring’ are not specific for one type of SLE.

Basis of the pre to post I comparison were 20 semiological features contained in the videos (compare Tables S2 and S3 in the Additional file 1: material). True positive, false positive and false negative documentation rates for semiological features were calculated. True negative rates could not be determined since study participants were not provided with a list of semiological features to choose from, and without such a list a virtually infinite number of features would be possible.

Positive predictive values (PPV) for individual semiological features were compared between pre and post I to examine the effect of training on recognizing semiological features correctly.

We further performed a correlation analysis using Spearman’s rank correlation coefficient to determine the relationship between the experience of the participants and their number of correct diagnoses pre or post training. The experience of participants was measured with three different features: years since obtaining medical degree, years as emergency physician and estimated number of patients with SLE treated in emergency settings (see tables S4.1 and S4.2).

## Results

40 out of 120 course attendees consented to be study participants and performed the pre and post I evaluation. Of 21 participants who agreed to participate also in the post II round, only 8 ultimately evaluated their third set of videos. All others indicated that lack of time was the reason for non-participation at post II.

### Work biographical data

For work-biographical data see Table 1.

**Table 1:** Work biographical data of the 40 study participants.

Variable	n	%
*Primary Specialty*		
Internist	7	17.5
Surgeon	9	22.5
Anesthetist	24	60.0
Else	0	0
*epileptological "experience"/training*		
Yes	1	2.5
No	39	97.5
*Experience as emergency physician*		
No experience^1^	2	5.0
< 1 year	7	17.5
1–3 years	11	27.5
4–6 years	10	25.0
≥ 6 years	10	25.0
*Number of seizures treated as emergency physician*^*2*^		
0	2	5.0
< 20	21	52.5
≥ 20	17	42.5
*Years since obtaining medical degree*		
Mean	8	
Range	3 to 20	
Median	6.75	

^1^ Participants with no experience underwent first time emergency physician training, all others attended the lesson as part of their continuous medical education. ^2^ Self estimate by the participant

### Accuracy of diagnoses pre course and post I

Prior to the lesson, 44.5% of 200 SLE ratings were correct. The diagnostic accuracy for ES (*n* = 120) was 59.2%, (for FBTCS (*n* = 40) 87.5%, and for FIAS (*n* = 80) 45%), for PNES (*n* = 40) 30%, and for SY (*n* = 40) 15%.

**Fig. 2:**
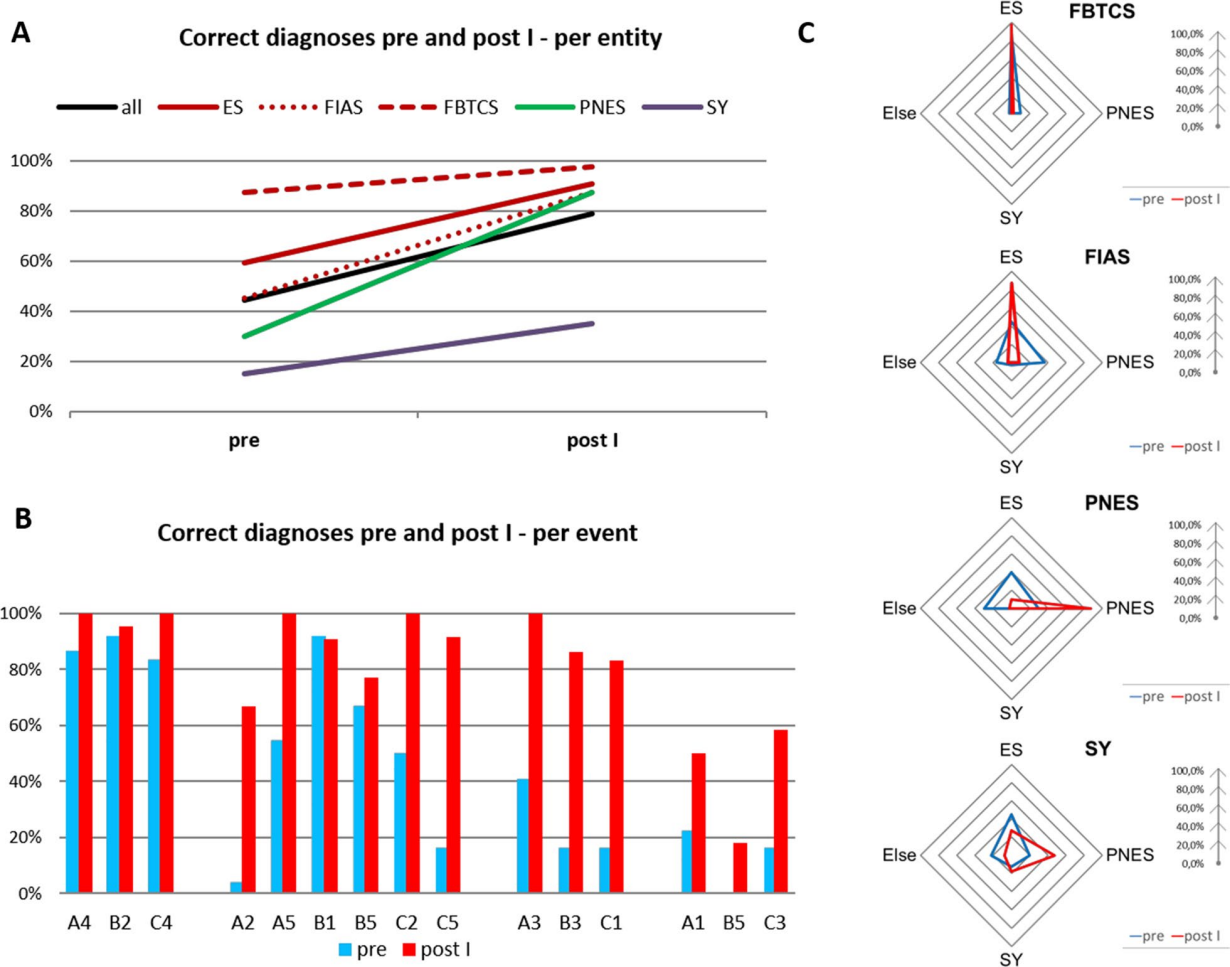
**A** Percentage of correct diagnoses of ES, PNES and SY at time points pre and post I. For each event type except FBTCS and SY, a significant increase was observed. **B** Also at the single event level, an increase in correct diagnoses is given in 14 out of 15 events (only FIAS B1 with ictal speech, prominent manual and subtle oral automatisms was correctly rated before the course in 91.7% and at post I in 90.9%). SY remain misdiagnosed at post I compared to ES and PNES. **C** Radar plots show participant’s diagnoses at time point pre (blue) and post I (red), ES are split up into FIAS and FBTCS. Pre-course FIAS, PNES and in particular SY showed a more diverse attribution of SLE (= larger area under the curve) including the diagnosis “other” (= no ES, PNES or SY) than FBTCS. After the course, FIAS, FBTCS and PNES were more consistently diagnosed correctly. In SY, the number of correct diagnoses increased, too, but the rate of misdiagnoses remained high

At post I, increased accuracy was observed for all event types (total 79% (*p* < 0.001) (ES overall 90.8% (*p* < 0.001), FBCTS 97.5% (n.s.), FIAS 87.5% (*p* < 0.001), PNES 87.5% (*p* < 0.001), SY 35% (*p* < 0.001)). Results are summarized in Fig. 2A.

The lesson thus significantly improved diagnostic accuracy across all SLE types except FBTCS, which were already well-recognized before the teaching (Table 2).

**Table 2:** Summary of training effect analysis before (pre) and immediately after the lesson (post I).

		Change of correctness from diagnoses from:		*p* value
	*n*	False pre to correct post I	Correct pre to false post I	
All events	200	77 (38.5%)	8 (4.0%)	< 0.001
ES	120	41 (34.2%)	3 (2.5%)	< 0.001
FIAS	80	37 (46.2%)	3 (3.8%)	< 0.001
FBTCS	40	4 (10.0%)	0 (0.0%)	n.s
PNES	40	24 (60.0%)	1 (2.5%)	< 0.001
SY	40	12 (30.0%)	4 (10.0%)	N.s

*p* value: McNemar’s Chi-squared test with continuity correction

Figure 2B shows an increase in correct diagnoses also at the individual event level except one FIAS. In ES, the range of pre-lesson correct diagnosis was 4.5 to 91.9%. The most frequently misdiagnosed events before the lesson were two FIAS (A2: 4.5%, an event presenting with ictal speech with partially remained responsiveness; and C5: 16.7% an event with receptive and expressive aphasia during testing). At post I, ES were correctly diagnosed between 66.7 and 100% (mean 91.3%). Events A2 and C5, were now diagnosed correctly by 66.7% and 91.7%, albeit by different study participants, see Fig. 1.

PNES were identified correctly before the lesson in 16.7% to 40.9%, and in 83.3% to 100% at post I.

Across SY, the pre-lesson correctness was 0% to 22.7%, at post I 18.2% to 58.3%.

### Analysis of false ratings

We analyzed if there was a tendency to confound ES, PNES and SY amongst each other or with other entities. Figure 2C shows that diagnoses were much more variable before the lesson (blue framed area), including the diagnosis “else” (which could be hypoglycemia, sleep disorder etc.) compared to post I (red framed area), except for SY.

### Persistence of learning effects (post II)

When comparing the performance of the 8 participants who completed all three video assessments over time, we found that the number of correct diagnoses increased at the post-II time point compared with pre-II, but decreased compared with post- I (see Table S3 in the Additional file 1). Due to the small number of participants at post II, no further analyses were performed.

### Documented semiological features

Analysis of documented semiological features was performed for the time points pre and post I and comprised all features listed in Additional file 1: Table S1. We considered semiological descriptions as correct which were sufficiently specific for the shown feature (e.g. tonic–clonic movement described as “patient stiff with rhythmic jerks” = correct; named as “shivering” = incorrect). By expert consensus (see methods), there were 4.4 distinguishing semiological features per video (ES 4.4, PNES 4, SY 4.67).

The number of semiological features documented by participants increased from 0.63 (14.3%) before to 1.37 (31.1%) per video after the lesson (post 1). An increase of documented semiological features was observed for all types of SLE: ES: 0.83 → 1.83 (18.9% → 41.6%), PNES 0.23 → 0.76 (5.8% → 19%), and SY 0.43 → 0.58 (9.2% → 12.4%).

Before the lesson, 75.4% of documented semiological features were correct (true positive, TP). At post I, 86.9% of all documented semiological features were TP. A substantial increase in TP was observed for PNES (22.2% before vs 90.3% after the lesson, and SY (29.4 before vs 91.3% after the lesson); the number of TP for ES was high before and after the lesson (88% and 85.9%).

In line with the increased number of documented semiological features and percentages of true positives on the group level, the PPV of several semiological features increased (see Table 3). Three semiological features characterizing PNES reached a PPV > 0.8 after the lesson (fluctuating course of the event, agonistic-antagonistic movement pattern, long event duration), two of them starting from a pre lesson PPV of 0.

**Table 3:**
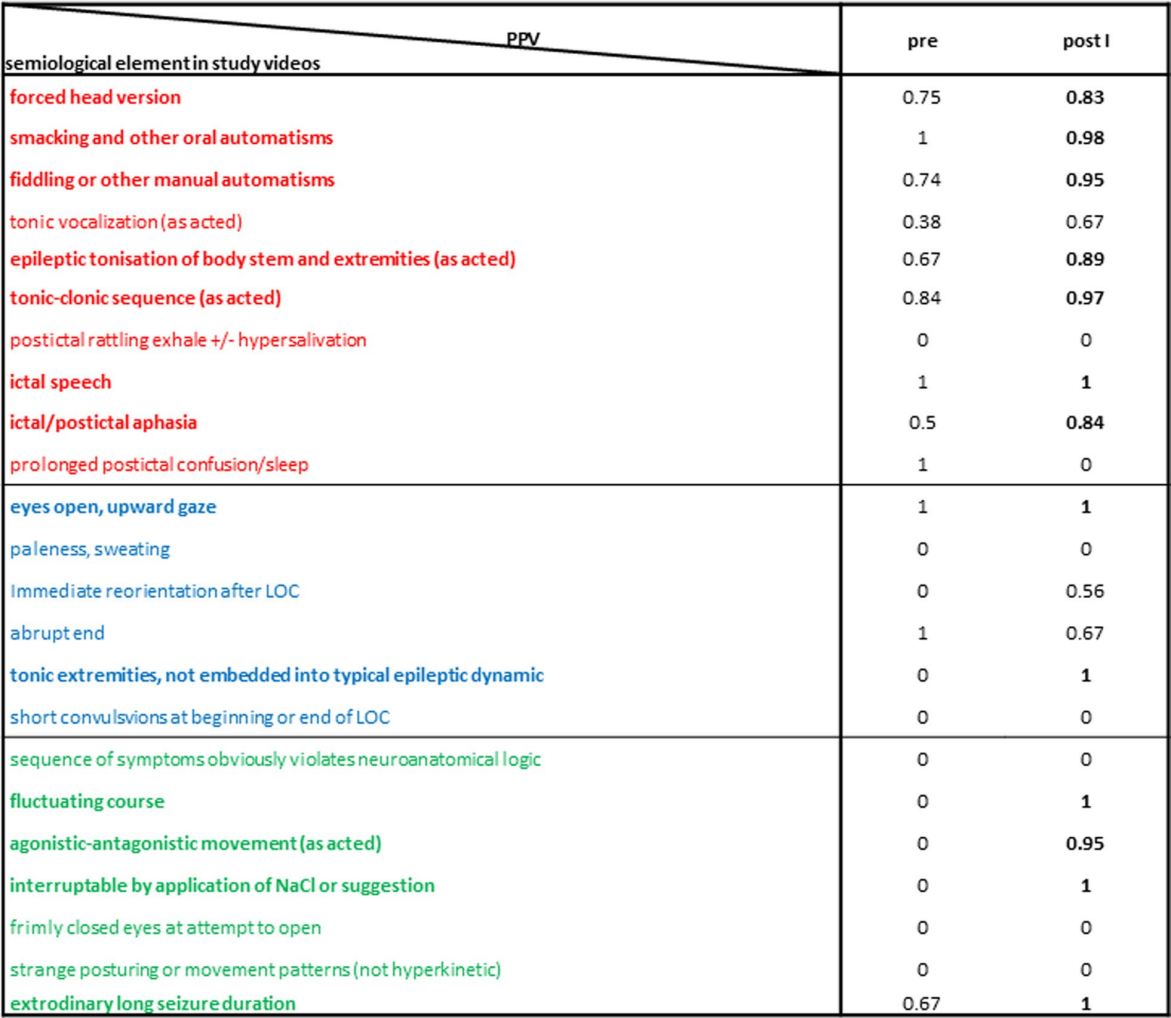
PPV of semiological features based on their listing in written reports

An increase in PPV indicates newly acquired knowledge. In contrast, a decreased PPV may indicate that under the given circumstances (only two minutes time to document the seizure) some semiological features were no longer regarded as important as other, newly learned features and therefore not documented. Semiological features with PPV > 0.8 bold printed.

In ES, many PPV were already high pre lesson. Nevertheless, teaching increased the PPV from below to over 0.8 for the following semiological features: ‘forced head deviation’ and ‘tonic stiffening embedded into epileptic dynamics’ in FBTCS, and ‘manual automatisms’ and ‘ictal and post-ictal aphasia’ in FIAS. At post I, seven semiological features typical for ES reached PPV > 0.8.

In SY, the PPV of “tonic body and extremities not embedded into typical epileptic dynamics” showed an increase from 0 to 1. Only one more feature (eyes open, upward gaze) had a PPV > 0.8.

### Relationship between professional experience and number of correct diagnoses

The correlation analyses revealed no significant correlation between participants’ experience and the number of correct diagnoses pre or post I lesson (see Additional file 1: Tables S4.1 and S4.2 in the Additional file 1).

## Discussion

In our sample, the baseline ability of emergency physicians to differentiate ES, PNES and SY correctly was limited (44.5% correct diagnoses), although 50% had been practicing as emergency physicians for at least four years, and 42.5% reported that they had attended > 20 SLE during their service. This finding fits well with the experience of neurologists and epileptologists that misdiagnostic regarding ES, PNES, SY and other paroxysmal health disturbances are common [3, 7, 11, 14].

Teaching emergency staff semiological features that allow for differential diagnosis on the scene (either by direct observation or through active witness interviews) seems to be viable and effective. We found a significant increase of correct diagnoses of SLEs from prior- to immediately after the lesson. The limited data from 8 participants who performed a third evaluation 6–8 months after the course (see Limitations) suggests that the learning effect is at least partially enduring.

A particularly strong pre- to post lesson increase in diagnostic accuracy could be observed for PNES and for the FIAS-subset of ES (both to 87.5% (*p* < 0.001)). We documented only a small, non-significant increase for FBTCS, yet these were recognized as epileptic already before the lesson (correct diagnoses 87.5% pre, 97.5% post (n.s.)) most likely since they are understood as the prototype of ES.

Diagnostic accuracy was lowest for SY (15% before and 35% after the lesson respectively (n.s.). However, this may partially be due to difficulties in selecting appropriate videos for our study. In our archive, videos of syncope are rare. Only a few SY were detected during video-EEG monitoring. Because SY (unlike ES and PNES) occurs only once in many patients, witnesses are usually unprepared, and therefore a cell phone video is not recorded. In addition: in syncope B5 we omitted the causative blood draw from the video, in video A1 features such as pallor were difficult to detect, and syncope C3 occurred in the context of a suggestive seizure induction, which may have confused the study participants.

Nevertheless, with the exception of SY, the pre-lesson variance of diagnoses was replaced by rather homogenous, correct diagnoses after the lesson (see areas under the curve in radar plots in Fig. 2C).

The key to better diagnosis of SLE probably lies in improving the recognition of specific semiological features. Two different semiology-related measures increased together with the correct number of diagnoses: the number of documented semiological features per video (which argues for a more profound semiological analysis of the events), and the number of semiological features with a PPV > 0.8.

A particular aspect of our analysis is that for some semiological features we considered their “sequential environment”. For example, the feature “stiffening” per se is rather unspecific (can be observed in ES, PNES and SY), however, embedded in a sequence of anteceding and following features it may well have a distinguishing value: Tonic stiffening, anteceded by a forced head deviation and a sustained vocalization and followed by a tonic clonic phase with decremental clonus frequency and a prolonged period of sleep and reorientation, argues for an ES, whereas tonic stiffening preceded by loss of consciousness plus upward tilt of the eye bulbs and followed by short convulsions with rapid reorientation suggest SY.

Analysis of the sequence of symptoms within epileptic seizures is not new. It has been routinely applied in presugical work-up of epilepsy patients for decades [18].Yet, when the sequence of symptoms fits well with neuroanatomy, this cannot only be used to explain the onset and spread of ES in the context of the presurgical evaluation, but it also differentiates ES from PNES in which these “topical templates” do not exist – at least to current knowledge. In SY, typical sequences exist, too, but they differ from ES and from what is typical for PNES [24]. To our knowledge, consideration of semiological sequences has not yet been applied to studies for differentiating ES, PNES and SY. We are currently investigating this topic in further detail.

Presenting videos of characteristic SLE and explaining them has been described before as being helpful to improve the correct diagnosis of SLE. Enacting particularly important semiological features by the lecturer, however, has not yet been described to the best of our knowledge. It may be beneficial via a more memorable effect than simply orally presenting a list of disjunct features. In addition, in our epileptological experience, witnesses can recognize enacted movements as applicable or not to the habitual events of patients. This often allows a clear cut differentiation between PNES (agonistic-antagonistic movement) and FBTCS (forced head deviation followed by tonic posture and then tonic–clonic movement). We believe that enabling emergency staff to enact few key features to witnesses might help to clarify the diagnosis of SLE before admission to a hospital.

### Limitations

The number of videos assessed by each study participant was low with respect to the heterogeneous spectrum of FIAS and PNES.

Only one third of the emergency physicians who attended the course agreed to participate in the study. This could reflect a bias towards motivated and inquisitive attendees and therefore the study cohort cannot be considered representative of all emergency physicians.

The same may be true for the low number of participants in the post II study. However, according to communication with potential post II participants, the reason for non-participation was lack of time rather than lack of interest.

The small number of post II participants makes it reasonable not to overstate the results. This is especially true for trends over time. Nonetheless, the negative trend in correctly scored PNES indicates that future studies should examine the durability of learning success and, if necessary, suggest follow-up training.

The list of semiological features typical for ES, PNES and SY used here is an expert consensus of the authors of this study and not the result of a formal statistical discriminant analysis. To the best of our knowledge such studies, comprising all three SLE entities, do not yet exist. However, the lacking formal proof of global discriminating power of the semiological features applied here does not affect the primary endpoint, which is the benefit of video-based teaching of semiological features to emergency physicians. Yet, our PPV-analysis of single semiological features can be the starting point for further studies addressing this aspect.

Lastly, although the video samples were selected by expert consensus, there was no control group of epileptologists who rated the videos in the same setting as the participants.

## Conclusion

A single 45-min lesson using videos and enactments of key semiological characteristics improves the diagnostic accuracy of SLE amongst emergency physicians.

In conclusion, our study shows that baseline expertise of emergency physicians regarding classification of seizure-like events is low, and obviously unrelated to years of experience or primary specialty. However, a single 45-min seminar using videos and enactments of key semiological characteristics can improve the diagnostic accuracy. Future studies should investigate the durability of this learning effect as well as its impact on emergency seizure management.

## Supplementary Information

**Additional file 1.** Supplementary tables.

## Data Availability

The datasets supporting the conclusions of this article are available from the corresponding author on reasonable request.

## Abbreviations

ECG: Electrocardiography
EEG: Electroencephalography
ES: Epileptic seizures
FBTCS: Focal to bilateral tonic–clonic seizure
FIAS: Focal impaired awareness seizures
PNES: Psychogenic non-epileptic seizures
PPV: Positive predictive values
SLE: Seizure like events
SY: Syncope
