# Supplementary material for: Teaching distinguishing semiological features improves diagnostic accuracy of seizure-like events by emergency physicians
Source: Neurol Res Pract. 2022 Nov 14;4:56. doi: 10.1186/s42466-022-00220-w (PMC9661782; doi:10.1186/s42466-022-00220-w)
Supplement: Supplementary file 1 — Additional file 1. Supplementary tables. [file 42466_2022_220_MOESM1_ESM.docx]

**Supplemental material**


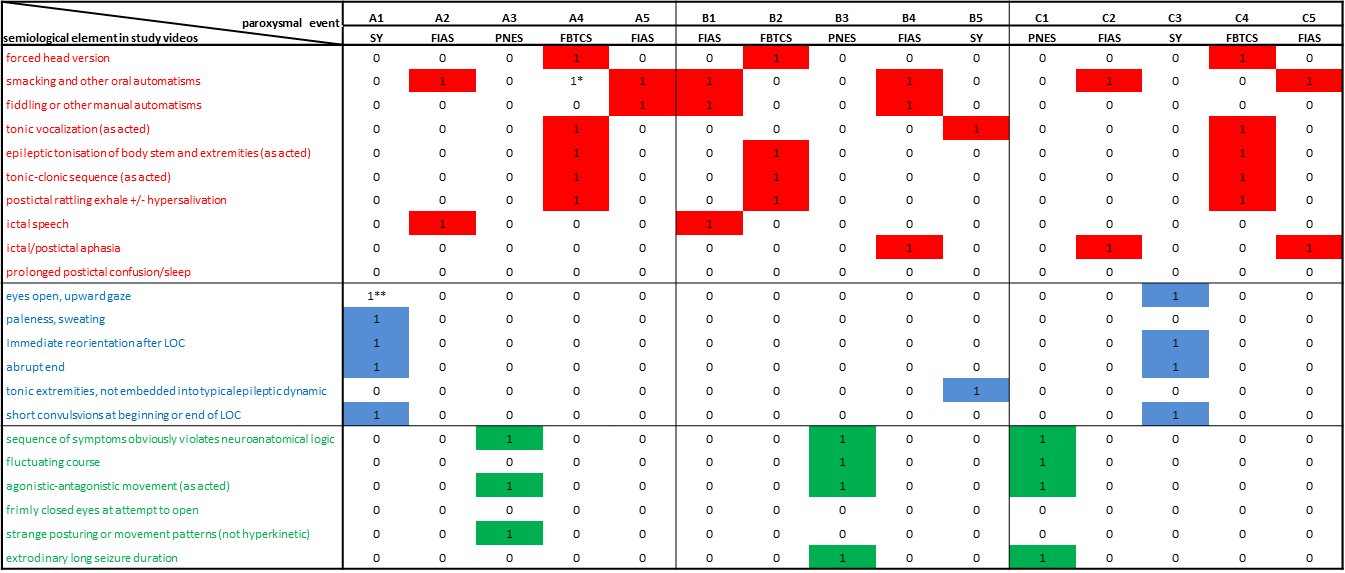


**Table S1:**

List of semiological features taught and, according to consensus assessment, contained in the 15 SLE videos shown in the study. Individual semiological features are not per se evidential for SLE etiology, but if all features of an event belong to the same color code (red = ES, blue = SY, green = PNES), the diagnosis should be possible with a high degree of certainty. A clear assignment of the listed events to only one entity was possible for 14 of 15 events, they contained only features of the same color. SY B5 contained features that could be assigned to both ES and SY. 1 = semiological feature present, 0 = not present in the respective event. * Feature not counted because seizure began with eating. ** Feature objectively present but not visible to participants due to pixelated face and therefore not counted. **Table S1** further specifies taught semiological features.


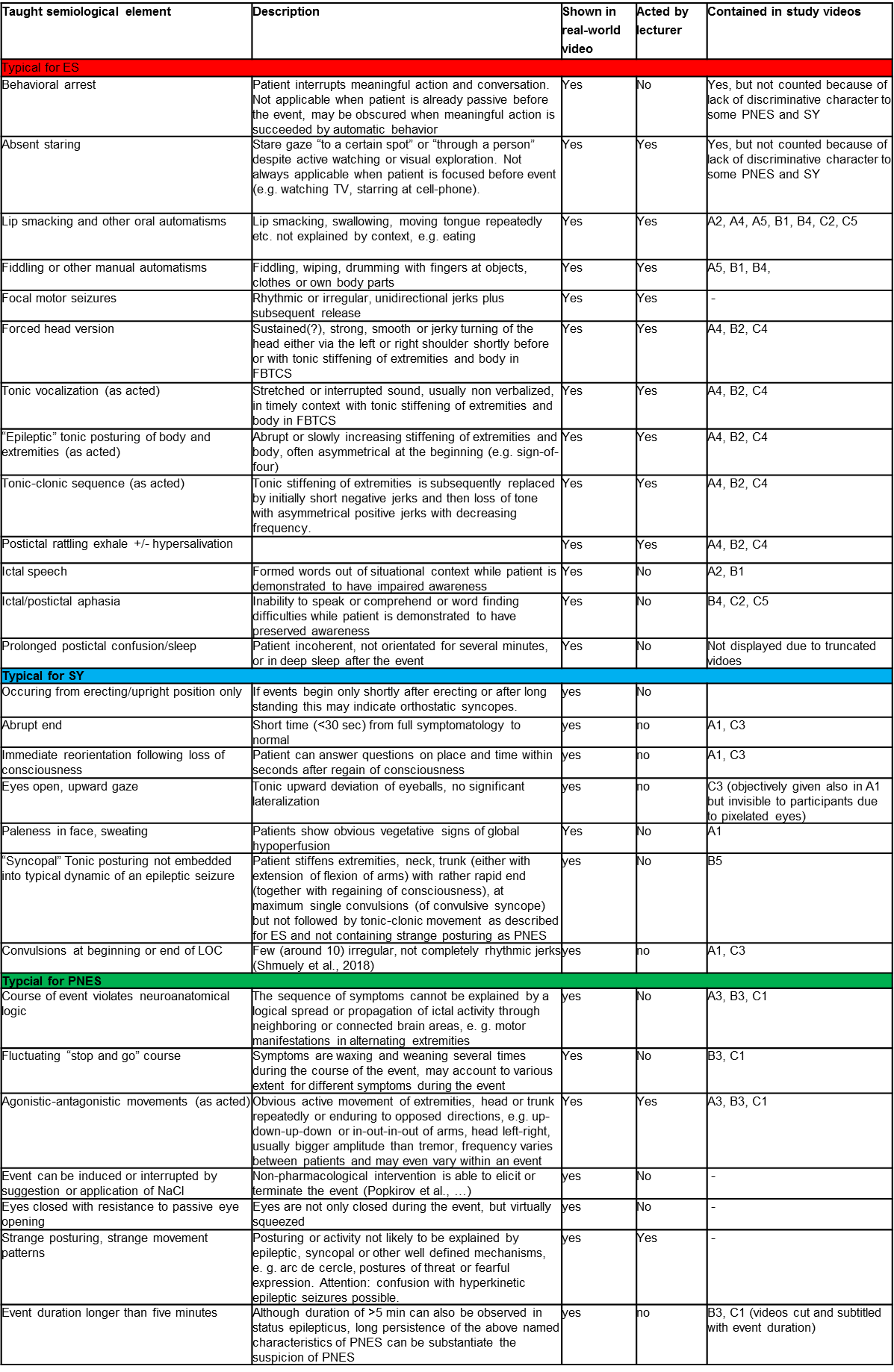


**Table S2:** Taught semiological features for differential diagnosis of ES, PNES and SY. This compilation reflects the personal experience of several authors from various international epilepsy centers and has been applied for clincal as well as teaching purposes.

|  |  | Correct diagnosis n (%) | | |
| --- | --- | --- | --- | --- |
|  | n | pre | post I | post II |
| All events | 40 | 15 (37.5%) | 32 (80.0%) (p<0.001) | 27 (67.5%) (n.s.) |
| ES | 24 | 13 (54.2%) | 22 (91.7%) (p=0,008) | 20 (83.3%) (n.s.) |
| FIAS | 16 | 6 (37.5%) | 15 (93.8%) (p=0,008) | 13 (81.2%) (n.s.) |
| FBTCS | 8 | 7 (87.5%) | 7 (87.5%) (n.s.) | 7 (87.5%) (n.s.) |
| PNES | 8 | 2 (25.0%) | 8 (100.0%) (n.s.) | 5 (62.5%) (n.s.) |
| SY | 8 | 0 (0.0%) | 2 (25.0%) (n.s.) | 2 (25.0%) (n.s.) |

**Table S3:** Effect size of teaching over time including post II (6-8 months after post I, N=8 study participants)

|  | p-value | SCC |
| --- | --- | --- |
| Years since they finished medical studies  Experience as emergency physician  Number of patients with seizures | 0.95  1.00  0.25 | 0,01  0,00  0,19 |

**Table S4.1**: Summary of correlation test number of correct diagnoses at pre training

SCC: Spearman`s rank correlation coefficient between variable and number of correct diagnoses pre-training.

|  | p-value | SCC |
| --- | --- | --- |
| Years since they finished medical studies  Experience as emergency physician  Number of patients with seizures | 0.45  0.73  0.71 | -0,12  0,06  -0,06 |

**Table S4.2.** summary correlation test number of correct diagnoses at post training

SCC: Spearman`s rank correlation coefficient between variable and number of correct diagnoses post-training.

The results in table S3.1 and S3.2 show that there is no significant correlation between the considered characteristics of the participants and the number of correct diagnoses in pre or post training
